# Supplementary material for: Minimal non-abelian nodal braiding in ideal metamaterials
Source: Nat Commun. 2023 Mar 6;14:1261. doi: 10.1038/s41467-023-36952-9 (PMC9988980; doi:10.1038/s41467-023-36952-9)
Supplement: Supplementary file 1 — Supplementary Information [file 41467_2023_36952_MOESM1_ESM.pdf]

SUPPLEMENTARY INFORMATION for

"Minimal non-abelian nodal braiding in ideal metamaterials"

Huahui Qiu,[1†] Qicheng Zhang,[1†] Tingzhi Liu,[1] Xiying Fan,[1] Fan Zhang,[2] and Chunyin Qiu,[1*]

[1]Key Laboratory of Artificial Micro- and Nano-Structures of Ministry of Education and School of Physics and Technology, Wuhan University, Wuhan 430072, China

[2]Department of Physics, University of Texas at Dallas, Richardson, Texas 75080, USA

Supplementary Note 1. Tight-binding model

The real-space Hamiltonian for the tight-binding model sketched in Fig. 1c can be expressed as

$$\mathcal{H}(\mathbf{r},t)=\sum_{i,j,\alpha}\omega_\alpha c_{i,j,\alpha}c_{i,j,\alpha}^\dagger+\kappa\sum_{i,j}\left(-c_{i,j,1}c_{i-1,j,2}^\dagger+c_{i,j,1}c_{i,j-1,2}^\dagger-c_{i,j,1}c_{i+1,j,3}^\dagger+c_{i,j,1}c_{i,j+1,3}^\dagger\right)$$

$$+\sum_{i,j}[2f(t)c_{i,j,2}c_{i,j,3}^\dagger+g(t)(c_{i,j,2}c_{i+1,j,3}^\dagger+c_{i,j,2}c_{i,j+1,3}^\dagger)]+\mathrm{H.c.} \qquad \text{(S1)}$$

where the indices i and j label the lattices along the x and y directions, ω_α denotes the onsite energy for the αth orbital (with $\alpha=1\sim3$) in a given unitcell, $c_{i,j,\alpha}^\dagger$ and $c_{i,j,\alpha}$ are the corresponding creation and annihilation operators, κ, $f(t)$ and $g(t)$ are coupling terms, and $\mathrm{H.c.}$ represents Hermitian conjugation as usual. Applying 2D Fourier transform to Eq. (S1), we obtain the braiding Hamiltonian in momentum space[1],

$$H(\mathbf{k},t)=\begin{pmatrix}\omega_1(t) & H_{12}(\mathbf{k}) & H_{12}^*(\mathbf{k})\\ H_{12}^*(\mathbf{k}) & \omega_2 & H_{23}(\mathbf{k},t)\\ H_{12}(\mathbf{k}) & H_{23}^*(\mathbf{k},t) & \omega_3\end{pmatrix}. \qquad \text{(S2)}$$

Specifically, the onsite energies $\omega_2=\omega_3=0$ for the orbitals 2 and 3, and the onsite energy of the orbital 1 is a function of the time t, i.e., $\omega_1(t)=\frac{\kappa}{2}(18-t-|t-2|)$; the hopping terms $H_{12}(\mathbf{k})=-\kappa e^{-ik_x}+\kappa e^{-ik_y}$ and $H_{23}(\mathbf{k},t)=2f(t)+g(t)(e^{ik_x}+e^{ik_y})$, where $f(t)=\frac{\kappa}{4}(-12+t+|t-8|)$ and $g(t)=\frac{\kappa}{2}(2+t-|t-2|)$. The coupling strength κ is a typical energy scale for the system, which is assumed to be 1 for simplicity. Therefore, our system can be characterized by the three t-dependent parameters, $\omega_1(t)$, $f(t)$, and $g(t)$, which are piecewise linear functions (Fig. 1d). In this work we focus on $t\in[0,10]$, within which an elegant but complete non-Abelian nodal braiding process can be realized, as shown in Supplementary Fig. 1 by the band structures plotted in the full 2D BZ for eight typical moments.

The symmetry of this elementary braiding Hamiltonian is described below. It includes time-reversal symmetry $T=K$ (complex conjugation), twofold rotation symmetry $C_{2z}=\mathbb{I}_1\oplus\sigma_x$, and their combination $C_{2z}T=(\mathbb{I}_1\oplus\sigma_x)K$. In addition, it features two mirrors, $\mathcal{M}_{11}=\mathbb{I}_1\oplus(-\sigma_x)$ and $\mathcal{M}_{1\bar{1}}=\mathbb{I}_1\oplus(-\sigma_0)$, with the subscripts 11 and $1\bar{1}$ denoting their normal directions, which enforce the motion of band nodes along the two mirror lines (Fig. 1f). Here σ_0 and σ_x are Pauli matrices, and $\mathbb{I}_1$ is a one-dimensional identity matrix. Owing to the $C_{2z}T$ symmetry, a real-valued symmetric Hamiltonian can be obtained by a unitary transformation, i.e., $H^R(\mathbf{k},t)=VH(\mathbf{k},t)V^\dagger$ with $V=\sqrt{\mathbb{I}_1\oplus\sigma_x}$, where the matrix elements $H_{11}^R=\omega_1(t)$, $H_{22}^R=-g(t)(\sin k_x+\sin k_y)$, $H_{33}^R=g(t)(\sin k_x+\sin k_y)$, $H_{12}^R=H_{21}^R=\kappa(-\cos k_x+\cos k_y+\sin k_x-\sin k_y)$, $H_{13}^R=H_{31}^R=\kappa(-\cos k_x+\cos k_y-\sin k_x+\sin k_y)$, and $H_{23}^R=H_{32}^R=2f(t)+g(t)(\cos k_x+$

$\cos k_y)$.

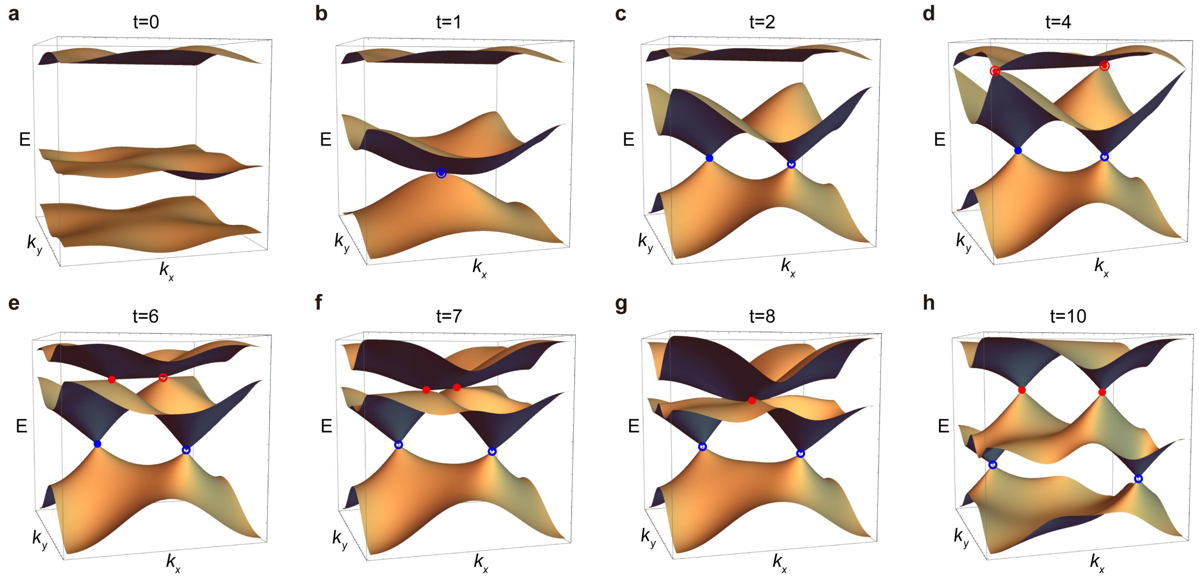

Supplementary Fig. 1 | Time evolution of the band nodes manifested by 2D full band structures. a-h, Band structures plotted in the full 2D BZ, which indicate the nontrivial nodal braiding in our main text. The whole process is very clean, which involves only two pairs of band nodes evolved along the HSLs of the square BZ. All results are calculated by the tight-binding model.

Supplementary Note 2. Non-Abelian topological charges for 2D band nodes and 1D gapped subsystems

Here we present details for determining the non-Abelian nodal charges, and point out that they can be directly related to those quaternion charges defined for 1D gapped subsystems of the original 2D Hamiltonian. To avoid confusion, here we define the QFC by Q for a band node in 2D and define the QFC by q for a 1D gapped three-band system. Importantly, resorting to the non-Abelian QFCs defined for 1D subsystems, we can explain the multi-gap topological edge states demonstrated in our main text (Fig. 5), whose evolution may serve as an edge manifestation of the non-Abelian braiding of band nodes.

The non-Abelian QFC for a band node is defined[2-6] as $Q = \exp[\oint \bar{\mathbf{A}}(\mathbf{k})\cdot d\mathbf{k}]$. Here $\bar{\mathbf{A}}(\mathbf{k}) = \boldsymbol{\beta}(\mathbf{k})\cdot(-\frac{i}{2}\boldsymbol{\sigma})$ is spin Berry-Wilczek-Zee connection in the spin(N)-valued 1-form, with $\boldsymbol{\sigma} = (\sigma_x, \sigma_y, \sigma_z)$ being Pauli matrices, $\boldsymbol{\beta}(\mathbf{k}) =- [\mathbf{A}_{23}(\mathbf{k}), \mathbf{A}_{31}(\mathbf{k}), \mathbf{A}_{12}(\mathbf{k})]$, and $\mathbf{A}_{mn}(\mathbf{k}) = \langle u_{\mathbf{k}}^m | \nabla_{\mathbf{k}} | u_{\mathbf{k}}^n \rangle$ being Berry-Wilczek-Zee connection. The subscripts m and n ($m, n = 1\sim3$) label the energy bands from the lowest to the highest, and $u_{\mathbf{k}}^n$ is an eigenstate of the nth band of the given Hamiltonian. The QFC values can be taken as $\pm\sigma_0$, $\mp i\sigma_x$, $\mp i\sigma_y$, and $\mp i\sigma_z$, which are respectively

represented by ±1, $\pm i$, $\pm j$, and $\pm k$ for simplicity. According to the multiplication rules of the Pauli matrices, the QFCs form the anticommutative quaternion group $G_Q = \{\pm1, \pm i, \pm j, \pm k\}$, in which the elements satisfy the fundamental multiplication rules $i^2 = j^2 = k^2 = -1$ and $ij = k, jk = i, ki = j$.

The QFC of a band node can also be directly inspected through the eigenstate trajectories plotted for a momentum loop encircling the node. More specifically, the trivial charge $Q = +1$ means that the three eigenstate trajectories can be continuously contractible to three isolated points. For the nontrivial charges $Q = \pm i$, the first eigenstate trajectory is continuously contractible to an isolated point, while the trajectories of the second and the third bands rotate by $\pm\pi$ with respect to the axis of the first band according to the right-hand rule; similar inspections can be used for the nontrivial charges $Q = \pm j$ and $Q = \pm k$. For the nontrivial QFC $Q = -1$, all the three eigenstate trajectories rotate by 2π. Note that the quaternion group G_Q can be classified into five conjugacy classes: $\{+1\}, \{\pm i\}, \{\pm j\}, \{\pm k\}, \{-1\}$. For a QFC belongs to the conjugacy classes $\{\pm i\}, \{\pm j\}, \{\pm k\}$, its sign is not well defined because of the gauge degree of freedom ±1 for the real-valued wavefunctions. For example, for a set of eigenstates $\{u_{\mathbf{k}}^1, u_{\mathbf{k}}^2, u_{\mathbf{k}}^3\}$ satisfying the right-hand rule (e.g., $u_{\mathbf{k}}^3 = u_{\mathbf{k}}^1 \times u_{\mathbf{k}}^2$), the sign of a QFC belonging to the three conjugacy classes changes if we flip the sign of $u_{\mathbf{k}}^1$ (or $u_{\mathbf{k}}^2$), i.e. $\{-u_{\mathbf{k}}^1, u_{\mathbf{k}}^2, -u_{\mathbf{k}}^3\}$ (or $\{u_{\mathbf{k}}^1, -u_{\mathbf{k}}^2, -u_{\mathbf{k}}^3\}$). Therefore, a pair of conjugate elements (e.g., $\pm i$) can be determined only when they share a common basepoint[2,3,5,6].

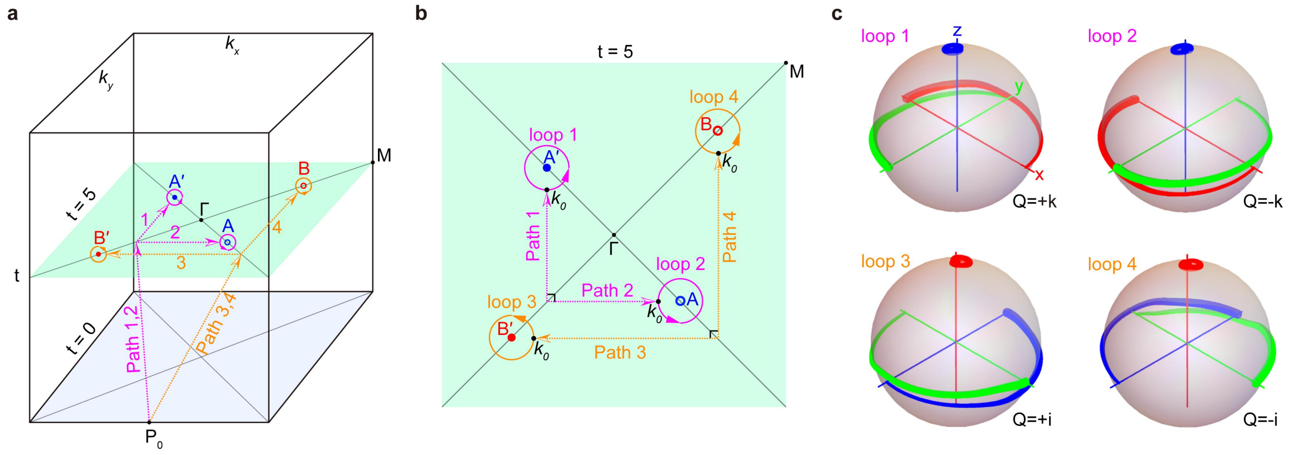

Supplementary Fig. 2 | Calculating QFCs for non-Abelian band nodes. a, Schematic illustration for calculating the QFCs of the two pairs of band nodes A(A') and B(B') exemplified at $t = 5$. The four circular loops (each encircling one node) are connected to the same basepoint P_0 through four different reference paths in the synthetic 3D reciprocal space (k_x, k_y, t). All paths bypass the node trajectories in the synthetic momentum space (see Fig. 1b), and each of them consists of two segments, one on the $t = 5$ plane and the other outside the plane. **b**, More details for the four

circular loops (with radii $k_r = 0.1\pi$) and the reference paths on the plane $t = 5$. **c**, Eigenstates frame spheres calculated for the four momentum loops, where the red, green, and blue curves represent the eigenstates $u_{\mathbf{k}}^1$, $u_{\mathbf{k}}^2$, and $u_{\mathbf{k}}^3$, respectively, and the curve from thin to thick depicts the anticlockwise orientation of each loop.

Now we demonstrate how the eigenstate trajectories can be calculated, which provides the time-dependent nodal QFC distributions in our main text (Fig. 1e). As sketched in Supplementary Fig. 2a, the combination of 2D square BZ and the parameter t forms a synthetic 3D reciprocal space (k_x, k_y, t). Take the moment $t = 5$ as an example, which hosts gap-I and gap-II nodes simultaneously. For each node, we consider an anticlockwise circular loop of radius 0.1π encircling it. To determine the sign of the nodal QFCs uniquely and universally for all t, we take the reference paths that connect the four circular loops to the same basepoint P_0 (with $k_x = 0$, $k_y = -\pi$, and $t = 0$) where the segments of the reference paths 1 and 2 (or the reference paths 3 and 4) on the k_x-k_y plane ($t = 5$) are selected to be perpendicular with each other (Supplementary Fig. 2b). The sign of each given eigenstate is selected such that a continue variation of the eigenstates is ensured along the whole reference path and the circular loop (i.e., $\mathrm{Re}\,\langle u_{\mathbf{k}}^n | u_{\mathbf{k}+\Delta\mathbf{k}}^n \rangle > 0$)[5]. Note that the reference paths can be arbitrarily selected as long as the nodes are avoided. Their selections may affect the moment when the charge sign is reversed, but will not change the underlying braiding physics. The reference paths selected here are representative: the QFC transition occurs when the gap-I and gap-II nodes share the same projections to the k_x-axis (also to the k_y-axis because of the two mirrors). Supplementary Figure 2c shows the eigenstates frame spheres calculated for the four momentum loops at $t = 5$, where the red, green, and blue curves respectively represent the trajectories of the eigenstates $u_{\mathbf{k}}^1$, $u_{\mathbf{k}}^2$, and $u_{\mathbf{k}}^3$ projected to those of starting-point $\mathbf{k}_0$, i.e., $\left(u_{\mathbf{k}_0}^1, u_{\mathbf{k}_0}^2, u_{\mathbf{k}_0}^3\right)^T \left(u_{\mathbf{k}}^1, u_{\mathbf{k}}^2, u_{\mathbf{k}}^3\right)$, and the curves from thin to thick depict the orientation of each loop[2-5]. Specifically, the eigenstates frame spheres give rise to the nodal QFCs $Q = \pm k$ and $\pm i$ for the two pairs of band nodes at $t = 5$.

Similarly, the QFCs can be defined for 1D gapped subsystems[3] of constant k_x. Supplementary Figure 3a sketches six 1D k_y-loops for calculating the loop QFCs, which bypass the band nodes and connect to the same basepoint P_0 through different reference paths in the synthetic 3D space (k_x, k_y, t). More specifically, the k_y-loop at $t = 0$ is defined by $k_x = 0$ and the five k_y-loops at $t = 7$ are defined by $k_x = -0.8\pi$, -0.38π, 0, 0.38π, and 0.8π, respectively. Supplementary Figure 3b shows eigenstates frame spheres calculated for the above six k_y-loops, which gives the loop

QFCs $q=+1$, $-i$, $+j$, $+k$, $-j$, and $-i$. Importantly, the loop QFC is closely related to the Zak phases of the three gapped bands at the same constant-k_x, and thus can be used to predict the presence of the multi-gap topological edge states[3]. (The case of $q=-1$ is an exception that goes beyond the Zak phase description. Fortunately, this case does not happen in our system.) Take $q=-i$ as an example, where the three bands (from the lowest to the highest) carry Zak phases 0, $-\pi$, and $-\pi$, respectively[3]. This explains the existence of gap-II edge states within the corresponding k_x intervals (see Figs. 5h-5k). We have calculated the k_x-dependent QFC distributions for the eight samples focused in our main text, which are consistent with the multi-gap edge states observed in Fig. 5.

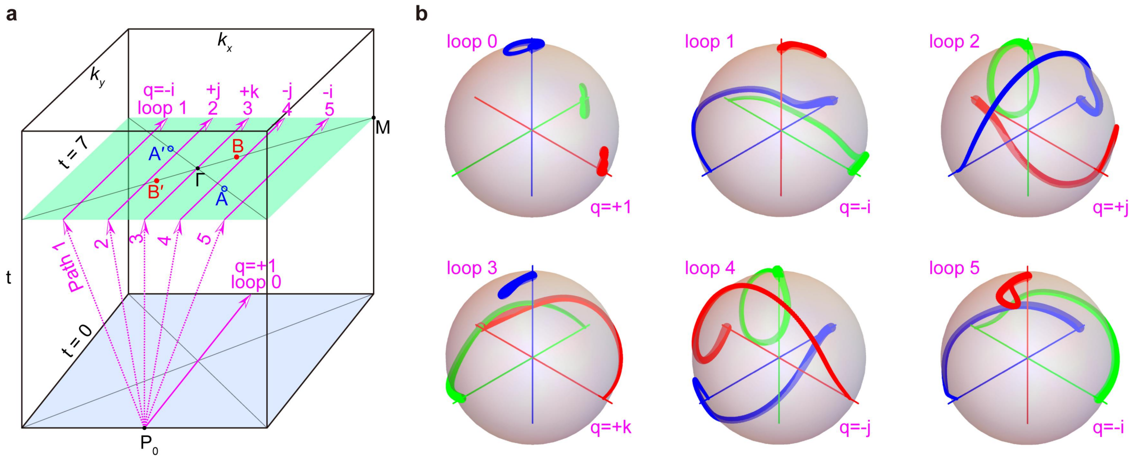

Supplementary Fig. 3 | Calculating QFCs for 1D subsystems with constant-k_x. **a**, Six 1D k_y-loops sketched for calculating QFCs, which bypass the band nodes and connect to the same basepoint P_0 through different reference paths in the synthetic 3D momentum space (k_x, k_y, t). More specifically, the k_y-loop at $t=0$ is defined by $k_x=0$ and the five k_y-loops at $t=7$ are defined by constant $k_x=-0.8\pi$, -0.38π, 0, 0.38π, and 0.8π, respectively. **b**, Eigenstates frame spheres calculated for the six k_y-loops defined in **a**, where the curves from thin to thick depict the orientation of each loop.

The nodal QFC (Q, defined by a closed loop encircling the node) is closely related to the QFCs of two 1D loops locating at the both sides of the node. As an example, we consider the gap-I node A' surrounded by the loops 1 and 2, which can continuously approach the band node without changing their QFCs[3]. As such, an infinitely narrow rectangular loop surrounding the node can be formed by the loop 2 (with QFC $q_2=+j$) plus a reversed loop 1 (with QFC $q_1^{-1}=+i$). The total rotation of

the eigenstates over the rectangular loop can be described by $\mathrm{Exp}(\pi L_y) \times \mathrm{Exp}(\pi L_x) = \mathrm{Exp}(\pi L_z)$, where $(L_i)_{jk} =- \epsilon_{ijk}$ with ϵ_{ijk} being the fully antisymmetric tensor. This gives rise to the QFC for the gap-I node A' $Q = q_2 \times q_1{}^{-1} = q_2/q_1 =- k$ according to a given sequential order[2-6]. Similarly, for an arbitrary given band node, its charge can be determined by $Q = q_r/q_l$, where q_r and q_l correspond to the QFCs defined for the 1D 1oops locating at the right and left sides of the node. Therefore, the topological edge states can reflect the multi-gap braiding effect of the band nodes.

Supplementary Note 3. Designing acoustic metamaterials

The tight-binding model can be emulated with our acoustic metamaterial made of cavity-tube structures in a square lattice (Supplementary Fig. 4a), where the cavity resonators and narrow tubes mimic respectively the atomic orbitals and hoppings between them. Specifically, each unitcell (Supplementary Fig. 4b) contains three air cavities connected by narrow tubes: the cavity 1 points to the (11) direction and the cavities 2 and 3 point to the $(1\overline{1})$ direction, whose centers are located at $(0,0,h_1)$ and $(\pm a/8, \pm a/8,0)$, respectively, with $h_1 = 9.4$ mm. Each cavity can support various resonance modes, whose frequencies depend on the cavity geometry. Here we consider the lowest dipole resonance mode polarized along the length direction, as circled in Supplementary Fig. 4c, whose frequency is inversely proportional to the cavity length. For simplicity, all the three cavities share a fixed cross section defined by $d_1 = 7.2$ mm and $d_2 = 8.0$ mm. To mimic the fixed onsite energies of the orbitals 2 and 3, $\omega_2 = \omega_3 = \omega_0$, the lengths of the cavities 2 and 3 are fixed to be $d_3 = 30.4$ mm; in contrast, the length of the cavity 1, defined by $L_\omega(t)$, is variable to simulate the time-dependent onsite energy of the orbital 1, $\omega_1(t)$. All the cavity geometries are carefully designed to enable our desired resonant frequencies far away from other cavity modes.

Now we turn to the coupling tubes, which have square cross sections for simplicity. The side lengths of the tubes 1, 2, and 3 are denoted by the constant $s = 1.84$ mm, and variables $L_f(t)$ and $L_g(t)$, respectively. Note that the strength of the acoustic hopping is (roughly) proportional to the cross-section area of the narrow tube while the sign of the hopping depends on the connectivity of the tube between the cavities[7-9]. We use three kinds of elementary coupling structures to simulate the hoppings $\pm\kappa$, $f(t)$, and $g(t)$ introduced for the tight-binding model, as demonstrated in Supplementary Figs. 4d-4f, respectively. Specifically, to realize the hoppings $\pm\kappa$ between the orbitals 1 and 2 (or 1 and 3), which feature the same strengths but different signs, the three cavities in configuration-I (Supplementary Fig. 4d) are connected by two straight tubes (#1) of identical geometries but different orientations (related with the mirror symmetry $\mathcal{M}_{1\overline{1}}$). To mimic the

(negative) intracell and (positive) intercell couplings between the orbitals 2 and 3, $f(t)$ and $g(t)$, different tube connectivities are considered between the cavities 2 and 3, as shown in configuration-II (Supplementary Fig. 4e) and configuration-III (Supplementary Fig. 4f), respectively. Note that in both configurations the coupling tubes (#2 and #3) are bent to V-shape for increasing their lengths and thus fine-tuning the central frequencies of the systems[9].

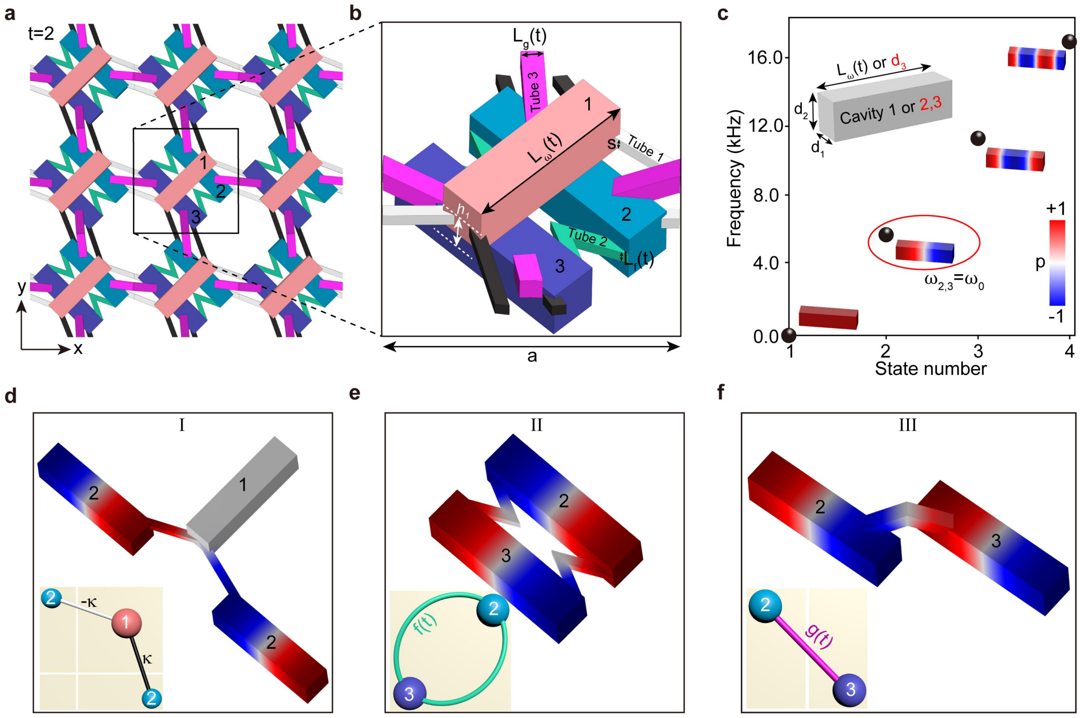

Supplementary Fig. 4 | Designing acoustic metamaterials that mimic the tight-binding model. a, Acoustic metamaterial made of cavity-tube structures in a square lattice. The lattice constant $a = 40$ mm. **b**, Unitcell geometry. **c**, Cavity modes of different resonant morphologies, where the fundamental dipole mode polarized along the length direction is highlighted. Inset: Cavity geometry. **d-f**, Three kinds of elementary structures used for realizing the desired couplings. The pressure field distribution exemplified for each configuration captures the corresponding eigenvector predicted by the tight-binding model (inset).

The effectiveness of the above three coupling structures can be checked by comparing their eigenstates with those predicted by tight-binding model. For conciseness, here we present only one representative eigenfield distribution for each configuration. Specifically, the eigenfield in Supplementary Fig. 4d corresponds to the middle eigenfrequency of the three-cavity system. The facts of zero pressure amplitude in the cavity 1 and the in-phase pressure distribution in the left two cavities are consistent with the eigenvector $(0,\frac{\sqrt{2}}{2},\frac{\sqrt{2}}{2})^T$ solved by the tight-binding model with

opposite couplings $\pm\kappa$. The eigenfield in Supplementary Fig. 4e corresponds to the higher eigenfrequency of the double-cavity system, where the out-of-phase pressure distribution in the two cavities reproduces the associated eigenvector $(\frac{\sqrt{2}}{2}, -\frac{\sqrt{2}}{2})^T$ of the tight-binding model with negative coupling $f(t)$. Similarly, the in-phase pressure distribution demonstrated in Supplementary Fig. 4f captures the eigenstate $(\frac{\sqrt{2}}{2}, \frac{\sqrt{2}}{2})^T$ of the tight-binding model with positive coupling $g(t)$. Combining the three kinds of elementary coupling structures, we obtain the unitcell (Supplementary Fig. 4b) that not only enables the positive and negative couplings but also meets the critical mirror symmetries in the model Hamiltonian. After a careful engineering process (see geometric parameters in Supplementary Tables 1 and 2), the band structures of our acoustic systems reproduce excellently those predicted by the tight-binding model (see Supplementary Fig. 5).

Supplementary Table 1. Position and orientation information of the coupling tubes.

Tube Number	Center Coordinate (mm)	Radial Vector
Tube 1	(16.81, 5.0, 4.70)	(1, -0.37, -0.47)
Tube 2	(4.07, -4.14, 0)	(1, -0.35, 0)
Tube 3	(0, 20.0, 7.6)	(1, -10, -6)

Supplementary Table 2. Unitcell geometries of the acoustic metamaterials.

Parameter	a	s	d_1	d_2	d_3	h_1	$L_\omega(t)$	$L_f(t)$	$L_g(t)$
Value (mm)	40.0	1.84	7.2	8.0	30.4	9.4	26.84~30.40	0~1.84	0~3.08

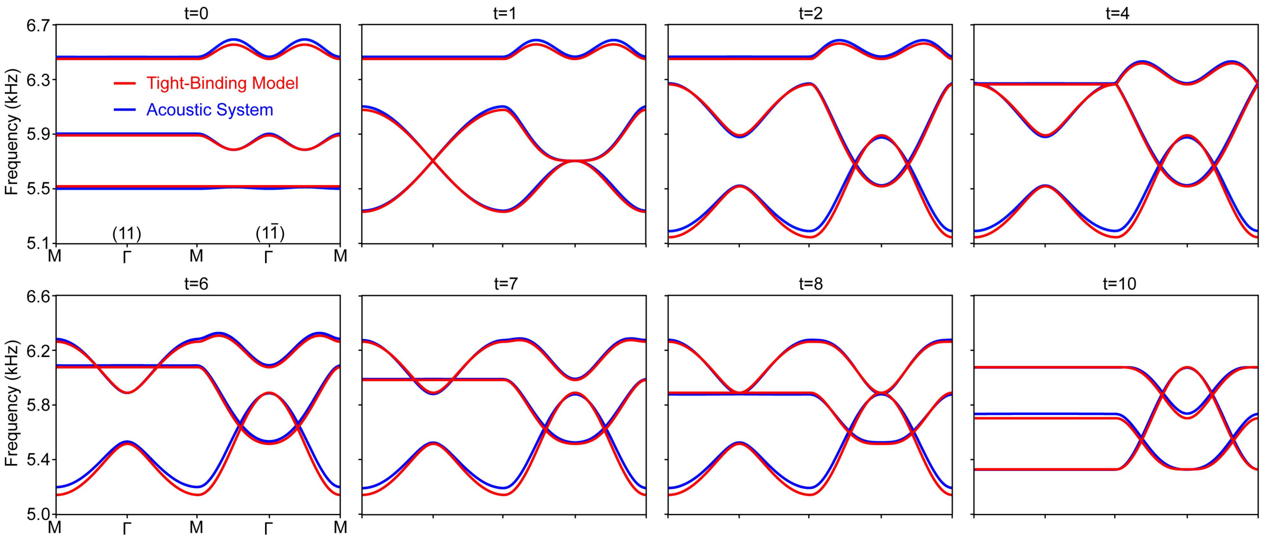

Supplementary Fig. 5 | Comparing the band structures between our acoustic system and tight-binding model. To fit the full-wave simulations, the coupling strength $\kappa = 93.5$ Hz and the onsite energy $\omega_0 = 5702.9$ Hz are consistently used in the tight-binding model.

Supplementary Note 4. Dispersion line shapes and stability of colliding nodes

Here we elaborate the relationship between the dispersion shape in the vicinity of the HSP node and the eigenvalues of the mirror operators $\mathcal{M}_{1\bar{1}}$ and $\mathcal{M}_{11}$, which is closely related to the stability of the HSP node. The conclusion is not limited to the tight-binding model used in Fig. 1c. Its generality can be proved as follows by a three-band $\mathbf{k}.\mathbf{p}$ effective model[10-12].

The component form of the effective Hamiltonian $H_{eff}(\mathbf{k})$ expanded to the second order can be expressed as

$$H_{mn} = c_1^{mn} + c_2^{mn} k_x + c_3^{mn} k_y + c_4^{mn} k_x^2 + c_5^{mn} k_y^2 + c_6^{mn} k_x k_y \tag{S3}$$

where $c_l^{mn} = a_l^{mn} + i b_l^{mn}$ ($m, n = 1\sim3$ and $l = 1\sim6$), i is the imaginary unit, a_l^{mn} and b_l^{mn} are real numbers, and $\mathbf{k} = (k_x, k_y)$ is Bloch wave vector measured from the HSP Γ or M. (The HSPs Γ and M feature the same symmetry and thus are not distinguished below.) Besides, the Hermiticity of the system requires $H_{mn} = H_{nm}^*$. Under the constraints of the time-reversal symmetry and the two mirror symmetries,

$$T H_{eff}(\mathbf{k}) T^{-1} = H_{eff}(-\mathbf{k}) \tag{S4}$$

$$\mathcal{M}_{1\bar{1}} H_{eff}(k_x, k_y) \mathcal{M}_{1\bar{1}}^{-1} = H_{eff}(k_y, k_x) \tag{S5}$$

$$\mathcal{M}_{11} H_{eff}(k_x, k_y) \mathcal{M}_{11}^{-1} = H_{eff}(-k_y, -k_x) \tag{S6}$$

$H_{eff}(\mathbf{k})$ can be simplified as

$$H_{11} = a_1^{11} + a_4^{11}(k_x^2 + k_y^2) + a_6^{11} k_x k_y \tag{S7}$$

$$H_{22} = a_1^{22} + a_4^{22}(k_x^2 + k_y^2) + a_6^{22} k_x k_y \tag{S8}$$

$$H_{12} = a_4^{12}\left(k_x^2 - k_y^2\right) + b_2^{12}\left(k_x - k_y\right)\mathrm{i} \tag{S9}$$

$$H_{23} = a_1^{23} + a_4^{23}\left(k_x^2 + k_y^2\right) + a_6^{23} k_x k_y + b_1^{23}\left(k_x + k_y\right)\mathrm{i} \tag{S10}$$

$$H_{33} = H_{22},\ H_{13} = H_{12}^*,\ H_{mn} = H_{nm}^*. \tag{S11}$$

Since the diagonal terms only affect the definition of zero energy, here we assume $a_1^{22} = 0$ for simplicity. From the eigenvalues and eigenstates of $H_{eff}(\mathbf{k})$, we can derive the information of the line shape around the HSP node and mirror eigenvalues of the HSP states. Specifically, for the HSP with $k_x = k_y = 0$, the energy eigenvalues are a_1^{11} and $\pm\, a_1^{23}$. This gives rise to three cases for the double degeneracy at the HSP, as listed below.

(**i**) $a_1^{23} = 0$ and $a_1^{11} \neq 0$. Along the $(1\bar{1})$ mirror line, the two energy bands around the HSP node behave as linear and carry identical mirror eigenvalues. Along the (11) mirror line, the two energy bands behave as quadratic and carry opposite mirror eigenvalues. This corresponds to the unstable gap-I node at Γ-point in our tight-binding model ($t = 1$).

(**ii**) $a_1^{11} =- a_1^{23} \neq 0$. Similar to the case (**i**), hybrid line shape can be inferred around the HSP node, but linear along the (11) direction and quadratic along the $(1\bar{1})$ direction, which feature respectively identical and opposite mirror eigenvalues. This corresponds to the unstable gap-II node at M-point in our tight-binding model ($t = 4$).

(**iii**) $a_1^{11} = a_1^{23} \neq 0$. The energy bands around the HSP node are quadratic in both mirror lines, and feature opposite eigenvalues for both mirrors. This corresponds to the stable gap-II node at Γ-point in our tight-binding model ($t = 8$).

A similar discussion can be made for the band node at a general point of the mirror line (11) or $(1\bar{1})$, where only one mirror constraint is imposed to the Hamiltonian. This gives the bilinear line shape around the node which belongs to the conjugacy classes $\{\pm i\}$ or $\{\pm k\}$ in our tight-binding model.

In summary, the dispersion shape around a HSP node is closely related to the mirror eigenvalues and thus to the stability of the HSP node. Therefore, we can experimentally demonstrate the HSP node stability either by inspecting the shape of dispersion curves around the node or by detecting the associated mirror eigenvalues.

Supplementary Note 5. Experimental details

5.1. Measurements of bulk and edge band structures

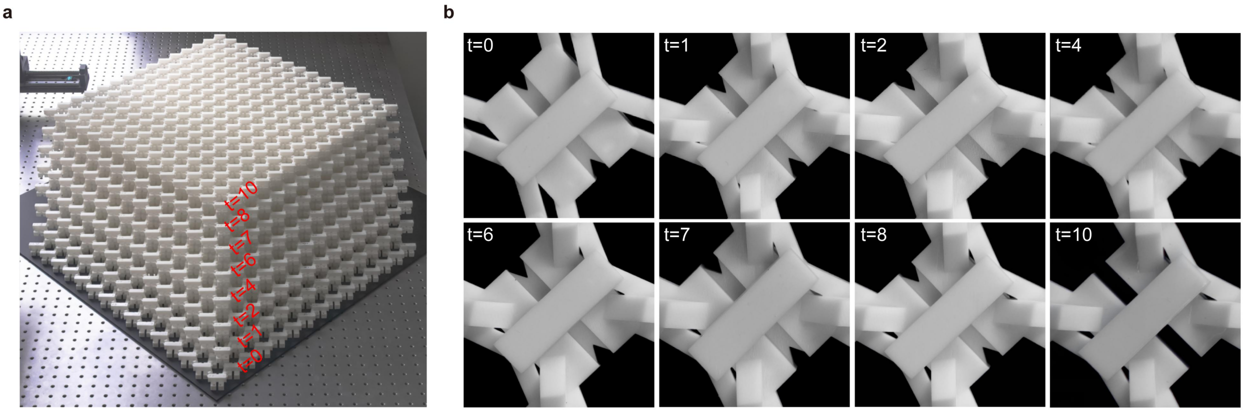

Supplementary Fig. 6 | Acoustic metamaterials fabricated for braiding multi-gap nodes. **a**, A photo taken for eight samples piled together. **b**, The associated unitcells.

Our acoustic metamaterials are fabricated with photosensitive resin through mature 3D printing technology. The air-filled cavities and coupling tubes are sealed with resin materials of thickness $1.4\ \mathrm{mm}$. Supplementary Figure 6a shows a photo for eight samples piled together. Each sample consists of $15 \times 15 = 225$ unitcells, associated with a spatial dimension of $600\ \mathrm{mm} \times 600\ \mathrm{mm} \times 20.2\ \mathrm{mm}$ in the x, y, and z directions. Supplementary Figure 6b shows the unitcell geometries for the eight samples, which exhibit observable differences in their fine structures.

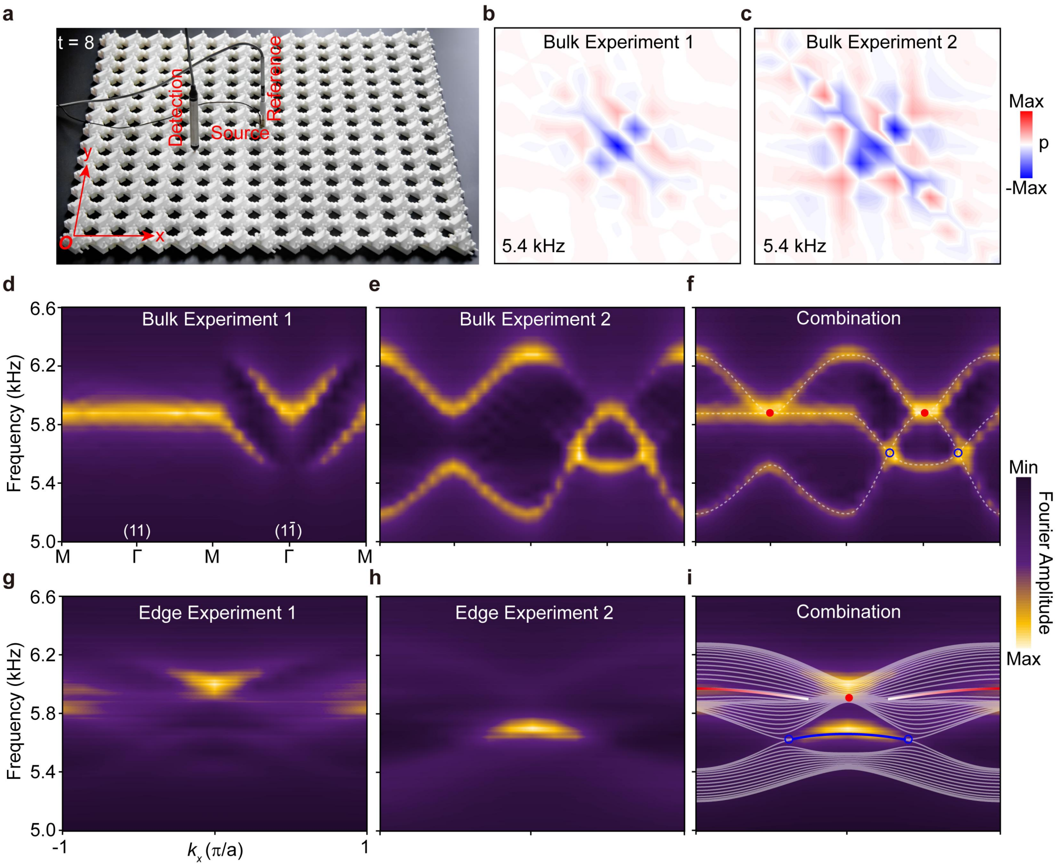

Supplementary Fig. 7 | Experimental measurements of the bulk and edge spectra (exemplified for $t = 8$). a, Experimental setup for our bulk measurements. The sound source is positioned in the middle unitcell of the sample, together with a microphone fixed in the same cavity for the phase reference. The pressure distribution over the sample is scanned by a needle microphone. Small holes are perforated on the desired cavities for inserting the sound source and detector, which are sealed when not in use. **b**,**c**, Pressure distributions measured independently for the cavities 1 and 2. **d**,**e**, The corresponding bulk spectra (color scale) plotted along the HSLs of the square BZ, obtained by performing 2D Fourier transforms to the above real-space measurements. **f**, Combination of the two independent measurements. The bright color reproduces perfectly the numerical dispersion curves (white dashed lines). **g-i**, Similar to **d-f**, but for the edge spectra, where the sound source and the reference microphone are relocated in the middle unitcell of the lower sample edge.

According to the theoretical analysis, the eigenstates are strongly polarized in three orbitals, especially for the states at the HSLs and HSPs, where the polarization between orbital 1 and the other two orbitals (2 and 3) are 100% (while the orbitals 2 and 3 are connected by mirror symmetry $\mathcal{M}_{11}$). This characteristic forces us to excite and detect in the cavities 1, 2, and 3 separately, so as to obtain all states as much as possible. As shown in Supplementary Fig. 7 (exemplified with $t = 8$), in our

bulk measurements we placed a loudspeaker in the cavity α ($\alpha = 1 \sim 3$) of the middle unitcell of the whole sample, fixed a microphone (B&K Type 4138) as the phase reference, and used a needle-like microphone (B&K Type 4182) to detect the pressure signal of all cavities α one by one. The frequency-resolved pressure distribution $p_i^\alpha(f)$ was obtained through frequency analysis by audio analyzer (B&K type 3560c), where the subscript i labels the unitcell and f is the frequency (at step of 8 Hz). In order to eliminate the influence of frequency-dependent excitation efficiency, we normalize the pressure field to the sound source, that is, $P_i^\alpha(f) = p_i^\alpha(f)/S(f)$, where $S(f)$ is the sound source signal in free space. Through 2D spatial Fourier transform, we obtain the spectrum $G_{\mathbf{k}}^\alpha(f)$ in momentum space. Further, we count all independent cavity measurements to obtain the bulk spectrum $G_{\mathbf{k}}(f) = \sqrt{\sum_{\alpha=1}^{3} |G_{\mathbf{k}}^\alpha(f)|^2}$. Supplementary Figures 7b and 7c exemplify the pressure distributions at 5.4 kHz for the independent measurements of the cavities 1 and 2. Supplementary Figures 7d and 7e we provide the corresponding Fourier spectra (color) plotted along the HSLs of the square BZ, which exhibit obvious sublattice polarizations (especially at HSPs). Supplementary Figure 7f gives the final bulk spectra (color scale), which reproduces perfectly our numerical simulation (white dashed line). Similar measurements were performed to obtain the edge spectra (Supplementary Figs. 7g-7i), where the sound source was relocated to the middle unitcell of the bottom edge of the sample. Note that the y-directed edges share the same physics with those x-directed edges, given the underlying mirror symmetries in our acoustic metamaterials.

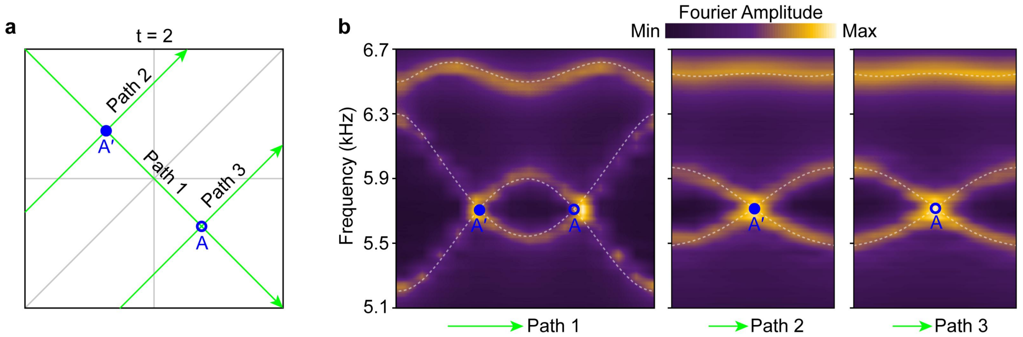

Supplementary Fig. 8 | Experimental characterization of the bilinear nodes (exemplified with $t = 2$). **a**, Momentum paths (green solid lines) selected for plotting the dispersion curves that connect two band nodes at general HSL momenta. **b**, Measured (bright color) and simulated (white dashed lines) bulk band structures along the momentum paths crossing at the gap-I nodes A and A', which together exhibit clearly bilinear behavior around the two nontrivial nodes.

As a supplement of Fig. 3 in our main text, here we present an experimental characterization of the

bilinear nodes. As shown in Supplementary Fig. 8, bilinear line shapes are demonstrated clearly in the spectra (Supplementary Figs. 8b and 8c) measured along the typical momentum paths sketched in Supplementary Fig. 8a.

5.2. Extracting HSP wavefunctions and expectation values of the mirror operators

From the 2D Fourier spectrum $G_{\mathbf{k}}^{\alpha}(f)$, we extracted the frequency-dependent wavefunctions at the HSPs, Γ and M. Specifically, the wavefunction $w_{\mathbf{k}}^{\alpha}(f_n) = G_{\mathbf{k}}^{\alpha}(f_n)/F$ was renormalized by a factor of $F = \sqrt{\sum_{n=1}^{N} |G_{\mathbf{k}}^{\alpha}(f_n)|^2}$ to enforce unitary sound energy over the spectra for the three independent excitations, i.e., $\sum_{n=1}^{N} |w_{\mathbf{k}}^{\alpha}(f_n)|^2 = 1$ ($\alpha = 1\sim3$), where the subscript n labels the discrete frequencies and $\mathbf{k}$ represents the HSPs Γ or M. Based on the wavefunctions, we further calculated the expectation value of the mirror operator $\mathcal{M} = \mathcal{M}_{1\bar{1}}$ or $\mathcal{M}_{11}$, according to $\overline{\mathcal{M}} = \langle \phi_{\mathbf{k}} | \mathcal{M} | \phi_{\mathbf{k}} \rangle$, where $|\phi_{\mathbf{k}}\rangle = (w_{\mathbf{k}}^1, w_{\mathbf{k}}^2, w_{\mathbf{k}}^3, -w_{\mathbf{k}}^1, -w_{\mathbf{k}}^2, -w_{\mathbf{k}}^3)^T / \sqrt{\sum_{\alpha=1}^{3} 2|w_{\mathbf{k}}^{\alpha}|^2}$ is the normalized wavefunction for a given frequency. Note that the information of the dipole-like cavity mode is considered here, and the mirror operators are expanded to 6×6 matrices accordingly. Detailed experimental data can be seen in Supplementary Fig. 9.

In order to further unveil the band inversion occurs during the time evolution, in Supplementary Fig. 10 we present the frequencies of the HSPs extracted from the experimentally measured (solid circles) band structures, together with the numerical (open circles) and theoretical (solid lines) results for comparisons. In particular, we encode the mirror expectation values for some typical moments. The band inversion, accompanying the evolution of bands with different mirror eigenvalues at the HSPs, is essential for changing the stability of the nodal pair in the same gap.

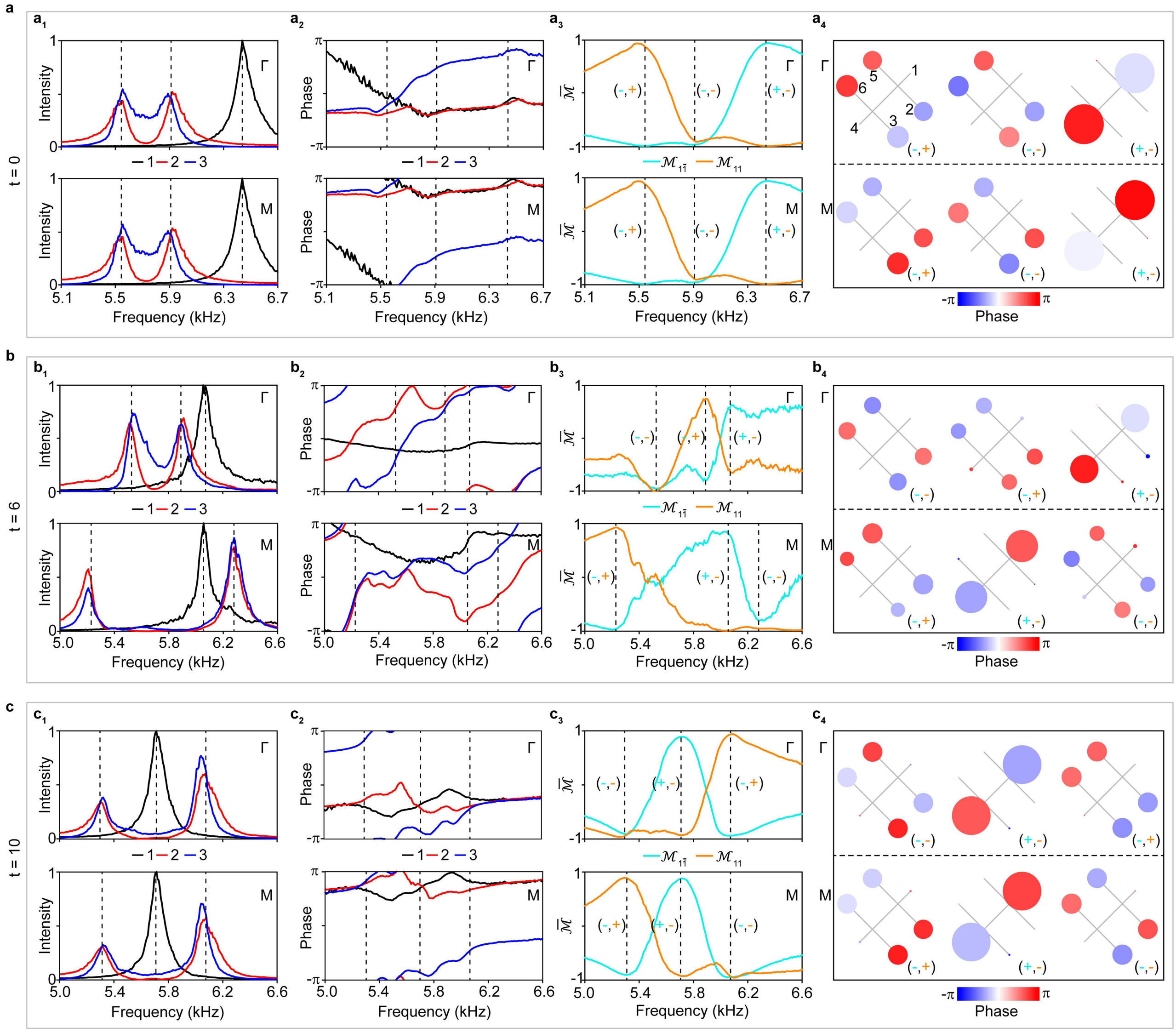

Supplementary Fig. 9 | Characterizing the HSP wavefunctions and their mirror symmetries. The experimental data in **a**-**c** correspond to three representative moments. **a$_1$**-**c$_1$**, Fourier intensity spectra extracted for the HSPs Γ and M, which are peaked precisely at the predicted eigenfrequencies (vertical dashed lines). The intensity peaks of the orbitals 2 and 3 (aligned because of the mirror symmetry $\mathcal{M}_{11}$) are misaligned with those of the orbital 1, which unveils the polarization nature of the HSP states. **a$_2$**-**c$_2$**, The corresponding phase spectra. **a$_3$**-**c$_3$**, Expectation value spectra of the mirror operators $\mathcal{M}_{1\bar{1}}$ and $\mathcal{M}_{11}$ measured for the wavefunctions at Γ and M. The mirror expectation value around each intensity peak approaches $+1$ or -1, which captures the mirror eigenvalue $+1$ or -1 for the symmetric or antisymmetric eigenstates. **a$_4$**-**c$_4$**, Field distributions extracted for directly visualizing the mirror symmetry of the HSP states. The sizes and color of the circles characterize the sound energy densities and phases of the detecting points, respectively. The frequencies used are labeled successively in **a$_1$**-**c$_1$** by the three dashed lines.

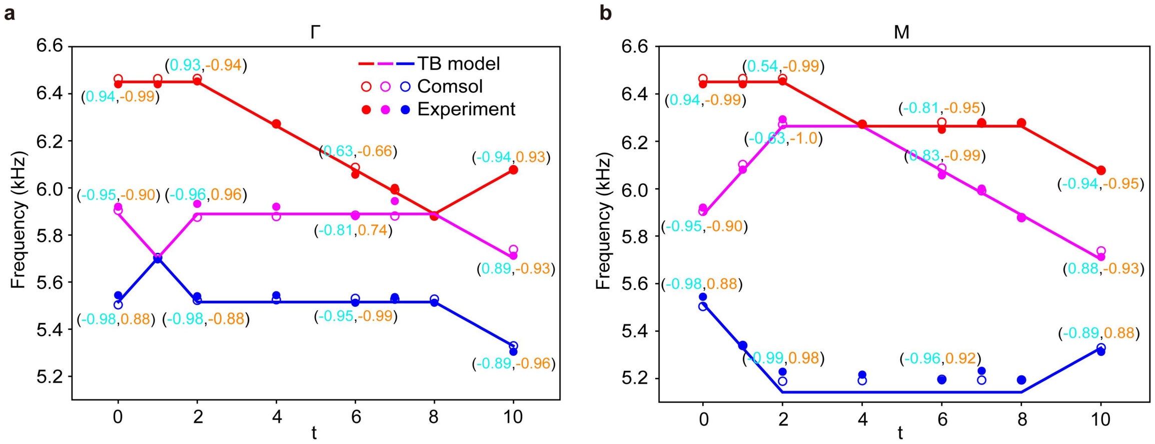

Supplementary Fig. 10 | Band inversions revealed by the time evolution of HSP states. a, Time involution of the Γ-point frequencies extracted from the experimentally measured (solid circles) band structures, together with the numerical (open circles) and theoretical (solid lines) results for comparisons. Mirror expectation values are labeled for the three moments $t = 0$, $t = 6$, and $t = 10$. **b**, The same as **a**, but for the M states. The band inversion, accompanying the evolution of bands with different mirror eigenvalues at the HSPs, is essential for changing the stability of the nodal pair in the same gap.

References

1. Bouhon, A., Wu, Q., Slager, R.-J., Weng, H., Yazyev, O.V. & Bzdušek, T. Non-Abelian reciprocal braiding of Weyl points and its manifestation in ZrTe. *Nat. Phys*. **16**, 1137-1143 (2020).

2. Wu, Q., Soluyanov, A. A. & Bzdušek, T. Non-Abelian band topology in noninteracting metals. *Science* **365**, 1273-1277 (2019).

3. Guo, Q. et al. Experimental observation of non-Abelian topological charges and edge states. *Nature* **594**, 195-200 (2021).

4. Yang, E. et al. Observation of non-Abelian nodal links in photonics. *Phys. Rev. Lett*. **125**, 033901 (2020).

5. Park, H. & Oh, S. S. Sign freedom of non-abelian topological charges in phononic and photonic topological semimetals. *New J. Phys*. **24**, 053042 (2022).

6. Tiwari, A. & Bzdušek, T. Non-Abelian topology of nodal-line rings in PT-symmetric systems. *Phys. Rev. B* **101**, 195130 (2020).

7. Xue, H., Yang, Y., Gao F., Chong, Y. & Zhang, B. Acoustic higher-order topological insulator on

a kagome lattice. *Nat. Mater.* **18**, 108-112 (2019).

8. Ni, X., Weiner, M., Alù, A. & Khanikaev, A. B. Observation of higher-order topological acoustic states protected by generalized chiral symmetry. *Nat. Mater.* **18**, 113-120 (2019).
9. Qi, Y., Qiu, C., Xiao, M., He, H., Ke, M. & Liu, Z. Acoustic Realization of Quadrupole Topological Insulators. *Phys. Rev. Lett.* **124**, 206601 (2020).
10. Lu, J., Qiu, C., Xu, S., Ye, Y., Ke, M. & Liu, Z. Dirac cones in two-dimensional artificial crystals for classical waves. *Phys. Rev. B* **89**, 134302 (2014).
11. Bradlyn, B., Cano, J., Wang, Z., Vergniory, M. G., Felser, C., Cava, R. J. & Bernevig, B. A. Beyond Dirac and Weyl fermions: Unconventional quasiparticles in conventional crystals, *Science* **353**, aaf5037 (2016).
12. Wu, W., Yu, Z., Zhou, X., Zhao, Y. X. & Yang, S. A. Higher order Dirac fermions in three dimensions, *Phys. Rev. B* **101**, 205134 (2020).
